# Supplementary material for: Chronic Adaptations to Eccentric Cycling Training: A Systematic Review and Meta-Analysis
Source: Int J Environ Res Public Health. 2023 Feb 6;20(4):2861. doi: 10.3390/ijerph20042861 (PMC9957439; doi:10.3390/ijerph20042861)
Supplement: Supplementary file 1 [file ijerph-20-02861-s001.zip › ijerph-2120327-supplementary.pdf]

Supplementary Material

Chronic Adaptations to Eccentric Cycling Training: A Systematic Review and Meta-Analysis

Renan Vieira Barreto*, Leonardo Coelho Rabello de Lima, Fernando Klitzke Borszcz, Ricardo Dantas de Lucas and Benedito Sérgio Denadai

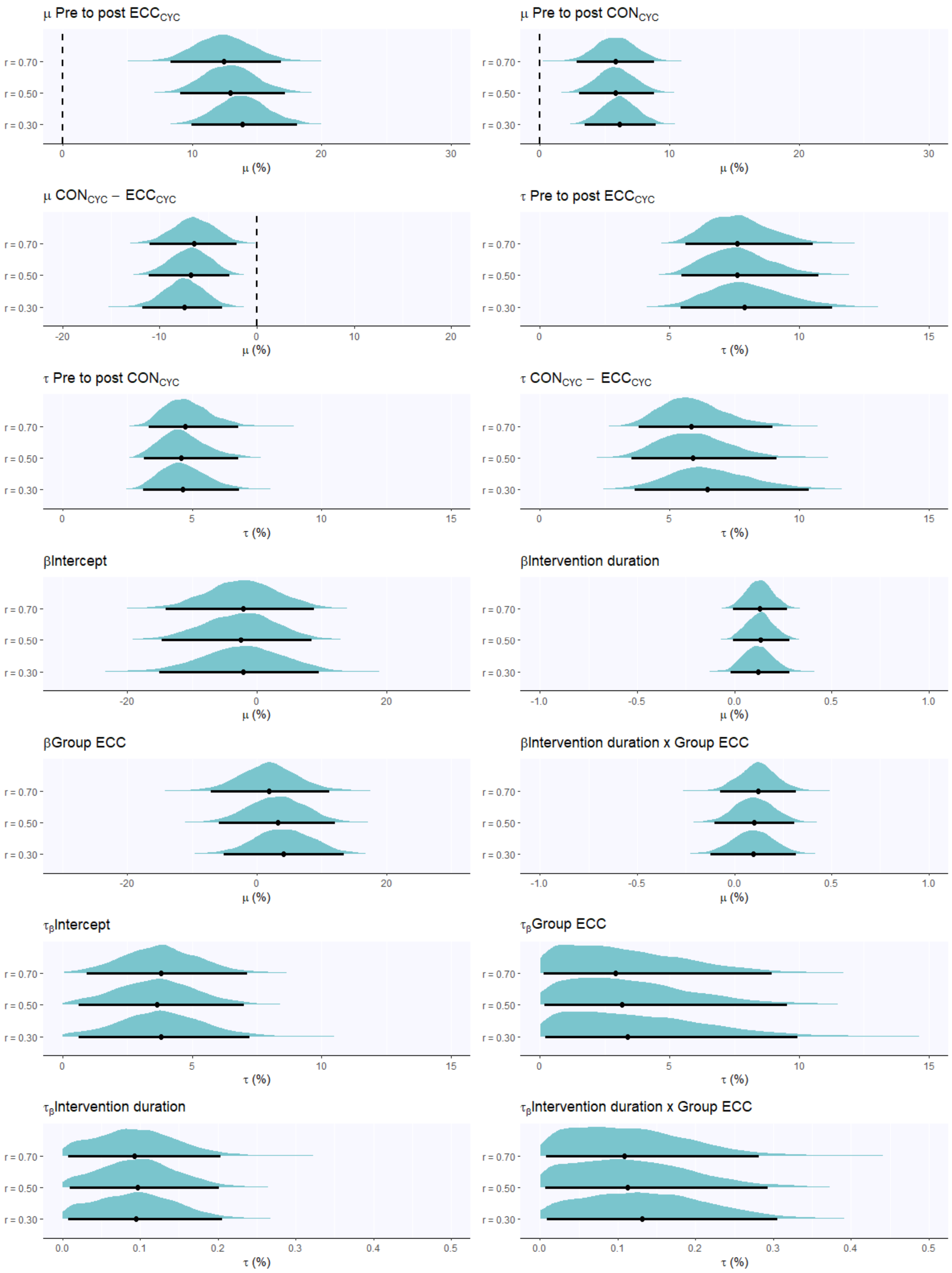

Figure S1: Sensitivity analysis results of different correlation coefficients (r = 0.30, 0.50, and 0.70) used in the variance-covariance matrix that addresses the dependence between the effect sizes derived from the same subjects for meta-

regression of eccentric cycling (ECC_{CYC}) training effects, concentric cycling (CON_{CYC}) training effects and net effects between modalities on isometric peak torque.

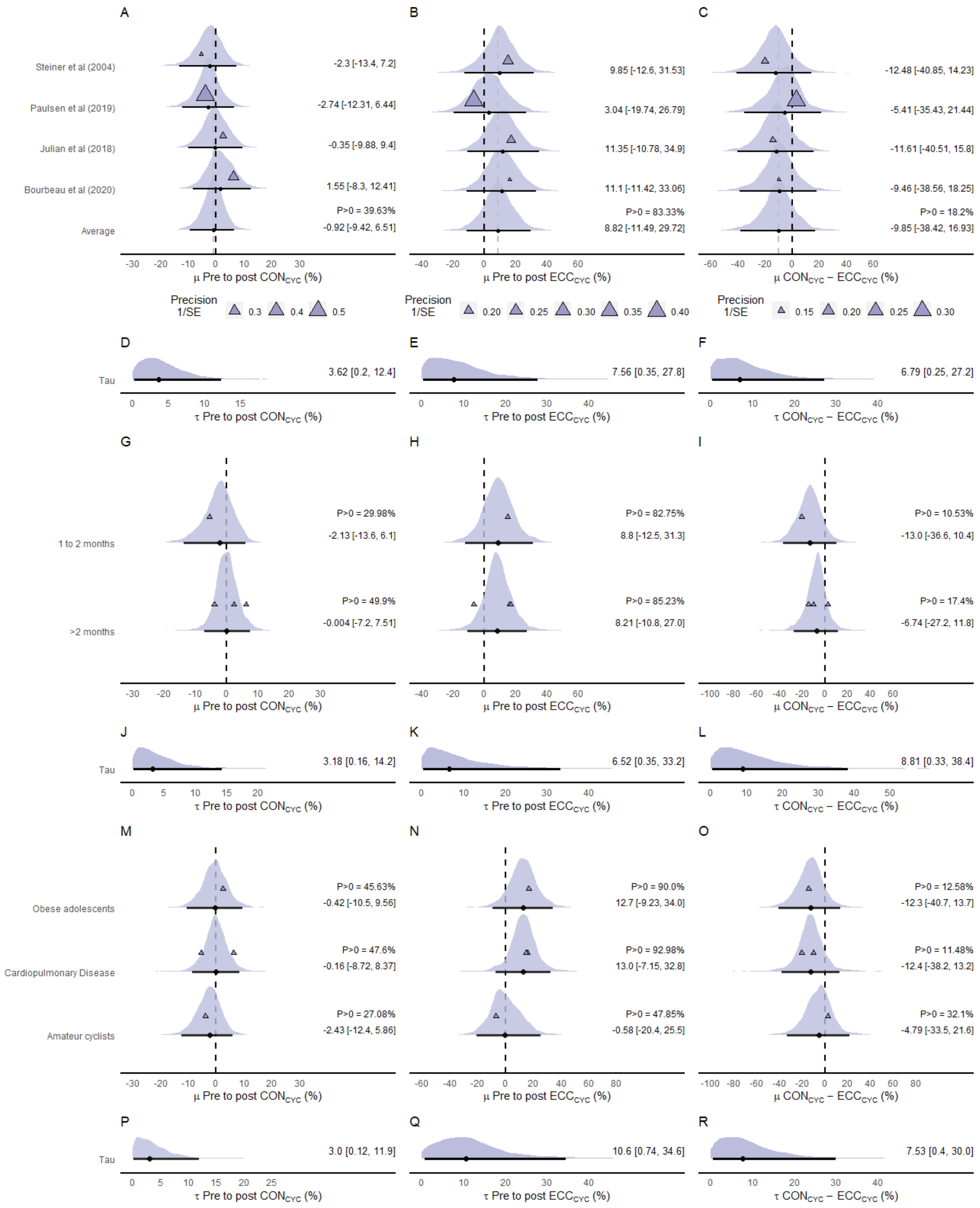

Figure S2: Forest plot of effect sizes (% mean difference) of CON_{CYC} training (a), ECC_{CYC} training (b), and net effect between training modalities (c) on isokinetic concentric peak torque. Heterogeneity between effects of CON_{CYC} training

(d), ECCcyc training (e), and net effects (f). Group-level effects of intervention duration of CONcyc training (g), ECCcyc training (h), and net effect between training modalities (i) on isokinetic concentric peak torque. Heterogeneity between group-level effects of intervention duration of CONcyc training (j), ECCcyc training (k), and net effects (l). Group-level effects of population of CONcyc training (m), ECCcyc training (n), and net effect between training modalities (o) on isokinetic concentric peak torque. Heterogeneity between group-level effects of population of CONcyc training (p), ECCcyc training (q), and net effects (r). The densities represent model estimates (i.e., the posterior distribution). Black dots and whiskers are the posterior effect size median and 95% credible interval, respectively. The triangles are the studies' observed mean effect sizes, and, in the panels a, b, and c, their sizes represent the precision of the effect, presented as the inverse of the standard error (1/SE), i.e., the larger the size of the triangle the smaller the standard error. Abbreviations: CONcyc – concentric cycling; ECCcyc – eccentric cycling.

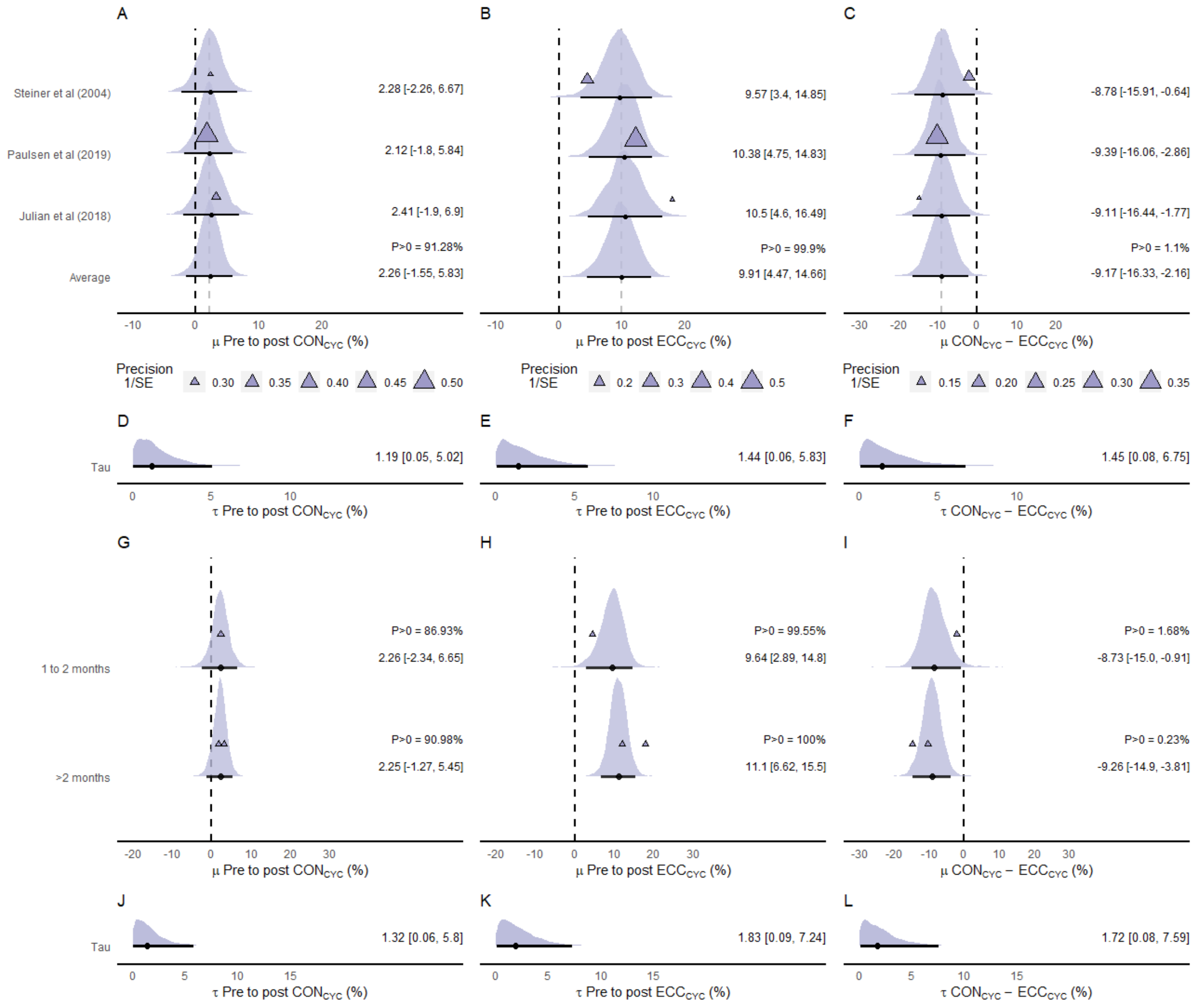

Figure S3: Forest plot of effect sizes (% mean difference) of CONcyc training (a), ECCcyc training (b), and net effect between training modalities (c) on isokinetic eccentric peak torque. Heterogeneity between effects of CONcyc training (d), ECCcyc training (e), and net effects (f). Group-level effects of intervention duration of CONcyc training (g), ECCcyc training (h), and net effect between training modalities (i) on isokinetic eccentric peak torque. Heterogeneity between

group-level effects of intervention duration of CONcyc training (j), ECCcyc training (k), and net effects (l). The densities represent model estimates (i.e., the posterior distribution). Black dots and whiskers are the posterior effect size median and 95% credible interval, respectively. The triangles are the studies' observed mean effect sizes, and, in the panels a, b, and c, their sizes represent the precision of the effect, presented as the inverse of the standard error (1/SE), i.e., the larger the size of the triangle the smaller the standard error. Abbreviations: CONcyc – concentric cycling; ECCcyc – eccentric cycling.

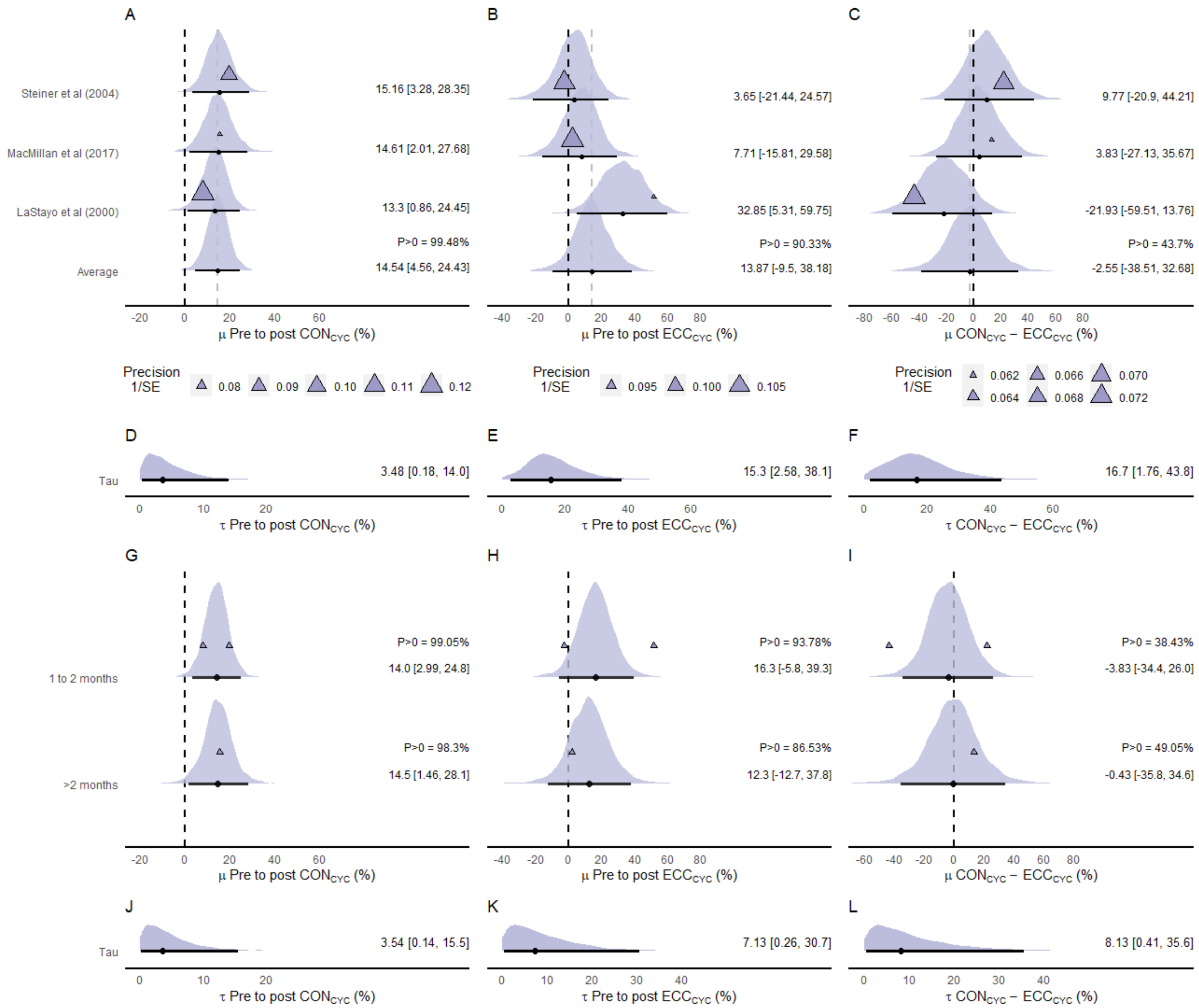

Figure S4: Forest plot of effect sizes (% mean difference) of CONcyc training (a), ECCcyc training (b), and net effect between training modalities (c) on fiber cross-sectional area. Heterogeneity between effects of CONcyc training (d), ECCcyc training (e), and net effects (f). Group-level effects of intervention duration of CONcyc training (g), ECCcyc training (h), and net effect between training modalities (i) on fiber cross-sectional area. Heterogeneity between group-level effects of intervention duration of CONcyc training (j), ECCcyc training (k), and net effects (l). The densities represent model estimates (i.e., the posterior distribution). Black dots and whiskers are the posterior effect size median

and 95% credible interval, respectively. The triangles are the studies' observed mean effect sizes, and, in the panels a, b, and c, their sizes represent the precision of the effect, presented as the inverse of the standard error (1/SE), i.e., the larger the size of the triangle the smaller the standard error. Abbreviations: CONCYC – concentric cycling; ECCCYC – eccentric cycling.

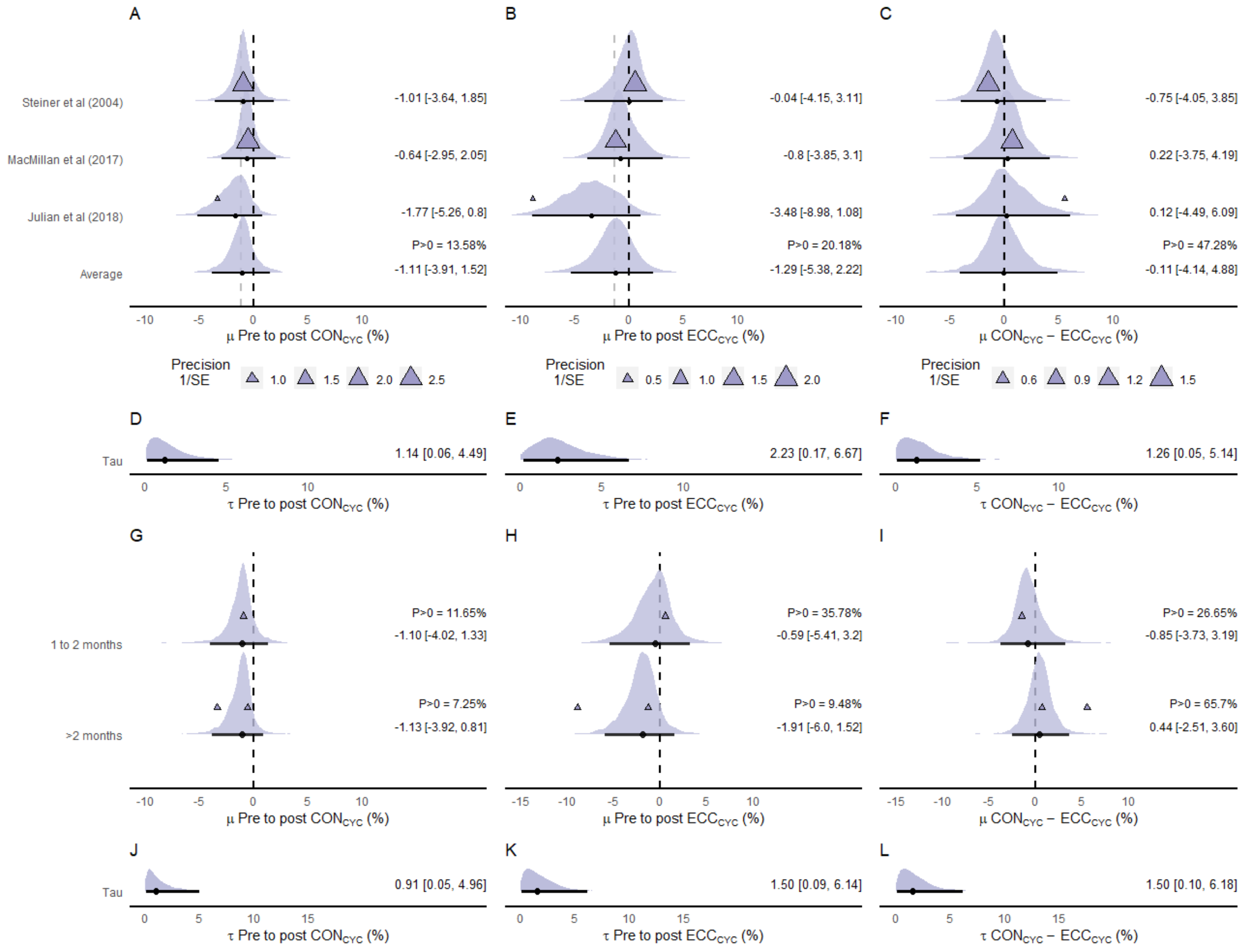

Figure S5: Forest plot of effect sizes (% mean difference) of CONCYC training (a), ECCCYC training (b), and net effect between training modalities (c) on body fat percentage. Heterogeneity between effects of CONCYC training (d), ECCCYC training (e), and net effects (f). Group-level effects of intervention duration of CONCYC training (g), ECCCYC training (h), and net effect between training modalities (i) on body fat percentage. Heterogeneity between group-level effects of intervention duration of CONCYC training (j), ECCCYC training (k), and net effects (l). The densities represent model estimates (i.e., the posterior distribution). Black dots and whiskers are the posterior effect size median and 95% credible interval, respectively. The triangles are the studies' observed mean effect sizes, and, in the panels a, b, and c, their sizes represent the precision of the effect, presented as the inverse of the standard error (1/SE), i.e., the larger the size of the triangle the smaller the standard error. Abbreviations: CONCYC – concentric cycling; ECCCYC – eccentric cycling.
